# Detection ability of model observer for diagnosis of active cardiac sarcoidosis on ^18^F-FDG-PET

**DOI:** 10.1007/s12149-026-02248-3

**Published:** 2026-06-15

**Authors:** Junichiro Naoi, Kentaro Takanami, Keisuke Kiso, Miho Shidahara, Hayato Odagiri, Daisuke Sakai, Takashi Noda, Satoshi Yasuda, Kei Takase

**Affiliations:** 1 Department of Diagnostic Radiology, Tohoku University Graduate School of Medicine, Sendai, Japan; 2 Radiology Center, Kindai University Hospital, Osaka, Japan; 3 Department of Diagnostic Radiology, Tohoku University Hospital, Sendai, Japan; 4 Division of Applied Quantum Medical Engineering, Department of Quantum Science and Energy Engineering, Graduate School of Engineering, Tohoku University, Sendai, Japan; 5 Department of Diagnostic Image Analysis, Tohoku University Graduate School o Medicine, Sendai, Japan; 6 Cardiovascular Center, Kindai University Hospital, Osaka, Japan; 7 Department of Cardiovascular Medicine, Tohoku University Graduate School of Medicine, Sendai, Japan

**Keywords:** PET, Cardiology, ^18^F-FDG, Sarcoidosis, Model Observer, CHO

## Abstract

**Objective:**

Sarcoidosis is an inflammatory granulomatous disease of unknown cause, and cardiac sarcoidosis (CS) is the main cause of death in patients with sarcoidosis. Cardiac ^18^F-Fluorodeoxyglucose (FDG)-positron emission tomography (PET) is used to detect active CS, based on the patterns of ^18^F-FDG uptake in the myocardium. In this study, we investigated the ability of a model observer, Channelized Hoteling Observer (CHO), in differentiating the active CS pattern, in comparison with the diagnosis by the expert cardiac nuclear medicine (NM) physician as a reference standard.

**Methods:**

This retrospective study included 223 patients who underwent ^18^F-FDG PET scans because they were clinically suspected of having CS. One expert cardiac NM physician and one oncology NM physician classified the cardiac ^18^F-FDG uptake into four patterns (focal, focal on diffuse, diffuse, none) for all PET images. The oncology NM physician additionally determined the confidence scale of CS expressed as a percentage (0-100). The focal and focal on diffuse patterns were defined as CS patterns. In CHO, 80 subjects (CS: 40, non-CS: 40) were used for training data sets, and 143 subjects (CS: 66, non-CS: 77) were used for evaluation data sets of objective CS discrimination ability, respectively. The confidence scale of CS by CHO was also output as a percentage (0-100). Finally, ROC analysis was used to compare discrimination ability between the oncology NM physician and CHO by refereeing the classification of the cardiac NM physician as a standard.

**Results:**

The match rate of classified subject numbers between the two physicians was moderate (Kappa coefficient of 0.57). In comparison with the judgment of the expert cardiac NM physician, the area under the ROC curve of oncology NM medicine and that of CHO were 0.73 and 0.78, respectively.

**Conclusions:**

The detection ability of CHO in CS pattern of myocardial ^18^F-FDG uptake was almost equivalent to that of the oncology NM physician when the diagnosis by the cardiac expert NM physician was defined as a reference standard. Even though the reference standard is not made by Japanese Circulation Society guidelines, the model observer has a potential alternative for assessing the discrimination ability of ^18^F-FDG uptake patterns of CS.

**Supplementary Information:**

The online version contains supplementary material available at https://doi.org/10.1007/s12149-026-02248-3.

## Introduction

Positron emission tomography (PET) enables quantifying the biodistribution of administrated radiopharmaceuticals in vivo [1, 2]. Recently, ^18^F-labelled fluorodeoxyglucose (^18^F-FDG) PET imaging has an important role in the clinical diagnosis of cardiac sarcoidosis (CS) [3–6]. Sarcoidosis is an inflammatory granulomatous disease of unknown cause, and CS is the main cause of death in patients with sarcoidosis [7]. Cardiac ^18^F-FDG PET is used for the detection of active CS, which demonstrates an increased ^18^F-FDG uptake in the myocardium. However, the diagnosis of CS is often difficult because physiological ^18^F-FDG uptake can also be observed in normal myocardium. For the differentiation, a visual assessment based on a four-pattern classification of myocardial ^18^F-FDG uptake, e.g. focal, focal on diffuse, diffuse, and none, can be used for the discrimination between the myocardial uptake due to CS and physiological myocardial uptake as well as other cardiac diseases such as dilated cardiomyopathy [4, 8]. However, the inter-observer agreement of cardiac ^18^F-FDG uptake image patterns was poor [9]. Furthermore, because of pattern-similarity, it is difficult to clinically discriminate two patterns, focal on diffuse and diffuse, for suspected patients of CS [8, 10]. Therefore, quantitative assessments of myocardial ^18^F-FDG uptake would be necessary for better discrimination [11–14].

Quantitative assessments by using a regional maximum of standard ^18^F-FDG uptake value in the myocardium (SUV_max_), cardiac metabolic volume (CMV), and cardiac metabolic activity (CMA) showed better discrimination than visual assessment [15, 16]. Recently, Manabe et al. reported that texture features in texture analysis, such as long runs emphasis (LRE), outperformed SUV_max_, CMV, and CMA when classifying suspected patients of CS [17]. Togo et al. introduced deep convolutional neural network (DCNN) and showed that image features derived from DCNN were more effective in the classification of cardiac sarcoidosis than conventional quantitative analysis such as SUV_max_ [9]. However, the discrimination ability of these previous approaches was assessed by only clinical diagnosis of CS [9, 15–17].

In general, clinical diagnosis of CS is based on the Japanese Society of Sarcoidosis and Other Granulomatous Disorders guideline (JSSOG), which originally includes not only the interpretation of PET images but also electrocardiogram, MRI, and so on [18]. The evaluation of PET image discrimination ability involves an inherent contradiction, as the test PET images have already been interpreted and diagnosed according to established guidelines, such as those of the JSSOG. Therefore, it is difficult to perform an ideal interpretation study to evaluate the pattern classification of ^18^F-FDG images for suspected patients of CS [19].

Recently, the numerical approach of signal detection in medical images, model observer, has been used for interpretation study instead of human observer [20, 21]. The advantages of the model observer are a high correlation of discrimination ability with the human observer in nuclear medicine, objective assessment by numerical analysis, and its easy implementation [22–25]. Even though it’s important, the use of model observers for the classification of PET images between CS-pattern and non-CS-pattern groups has not been reported yet. Therefore, in this study, we investigated whether the model observer, Channelized Hoteling Observer (CHO), can be an alternative approach against the human observer in the classification of PET images between CS-pattern and non-CS pattern groups, using the diagnosis of an expert cardiac nuclear medicine (NM) physician as a reference standard.

## Materials and methods

### Subjects

The subjects in this study were 245 patients who had cardiac ^18^F-FDG-PET/CT scans from Jan. 2015 to Dec. 2019 at Tohoku University Hospital because they were clinically suspected of having CS. 22 patients were excluded for reasons of less than 20 y.o. or a history of coronary artery disease. In this study, 223 PET/CT images were finally analyzed. Cardiac biopsy was performed in 126 of 223 patients, and histological confirmation was obtained in 23 patients (**Supplementary Table S2**). Because the number of clinically or histologically confirmed cases was limited, this study focused on diagnostic concordance rather than diagnostic accuracy. This study was approved by the ethical committee of Tohoku University Graduate School of Medicine (No. 2020-1-730).

### PET protocol

^18^F-FDG PET/CT images were obtained at our institution with a Biograph-40 PET/CT scanner (Siemens Healthcare, Erlangen, Germany). The patients were instructed to fast for at least 12 h before ^18^F-FDG injection. Approximately 3.7 MBq / kg body weight of ^18^F-FDG was injected through an antecubital vein. For the patients weighing over 90 kg, 333 MBq (9 mCi) of ^18^F-FDG was injected.

After approximately 1 h, a spiral CT scan was performed (with around 25 effective mAs, 120 kVp and a 2-mm slice thickness), followed by the collection of PET emission images from the chest to the upper abdomen. For 139 of 223 patients, thoracic PET was scanned in 3 bed positions.　The PET scanning time was 5 min per bed position with increments of 16.2 cm (3-dimensional mode) and reconstructed with 256 × 256 matrix (2.7 × 2.7 × 2.0). For 84 of 223 patients, whole-body PET was scanned in 8 bed positions and reconstructed with 168 × 168matrix (4.1 × 4.1 × 2.0). The PET scanning time was 2 min per bed position with increments of 16.2 cm (3-dimensional mode). PET images were reconstructed using iterative algorithms (ordered-subset expectation maximization, 6 iterations, 14 subsets). A 8-mm full-width at half maximum (FWHM) Gaussian filter was applied after the reconstruction. To match the matrix of the head and whole-body PET and that of the thoracic PET, whole-body images were resampled by trilinear interpolation to 256 × 256 matrix (2.7 × 2.7 × 2.0) by PMOD 3.1 (Pmod technologies LLC., Zurich, Switzerland).

### Image interpretation

To evaluate the visual discrimination ability of myocardial ^18^F-FDG uptake patterns in PET images, a slice-based interpretation study was performed by two physicians with a commercial image-viewer, EV Insite (PSP, Tokyo, Japan). In the routine diagnostic interpretation of ^18^F-FDG PET images, cardiac NM physician, with 26 years of clinical experience, specializes primarily in cardiac NM, whereas oncology NM physician, with 20 years of clinical experience, specializes mainly in oncology NM. As training before the evaluation, the two physicians completed a one-hour practice session on rating and classification using four PET images reported in a previous study [4].

**Fig. 1:**
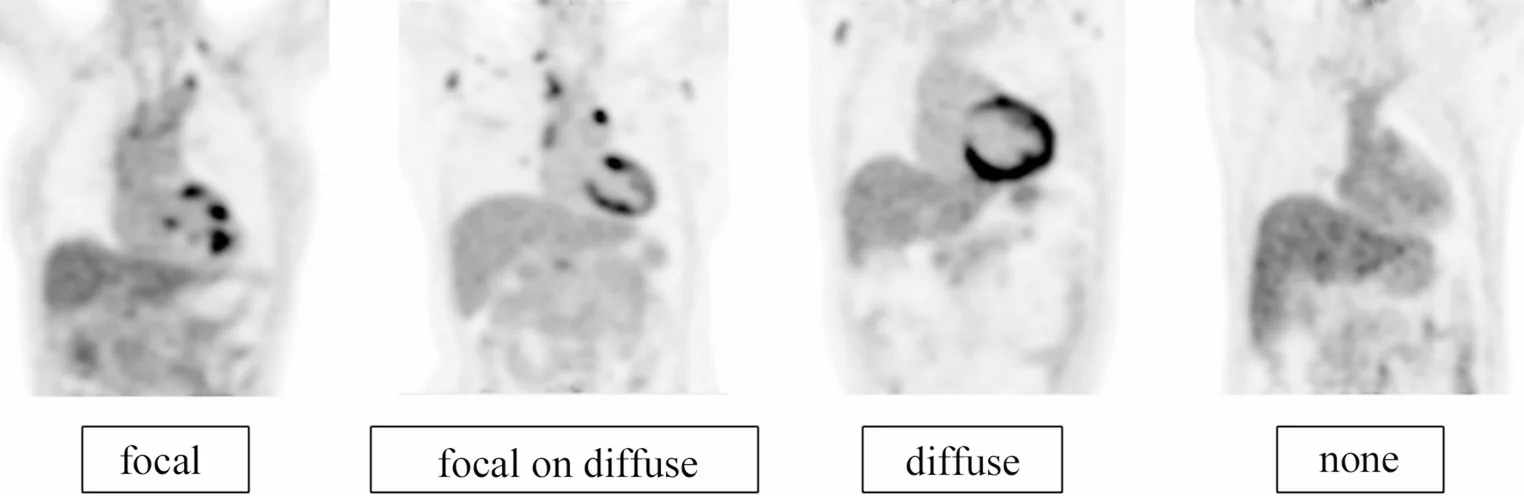
Examples of typical uptake patterns of ^18^F-FDG PET images

In this study, cardiac and oncology NM physicians performed a visual assessment of ^18^F-FDG uptake patterns for all PET images (*n* = 223), classifying them into CS patterns (focal and focal on diffuse) and non-CS patterns (diffuse and none) (Fig. 1) [4, 8]. The oncology NM physician additionally evaluated all PET images for the likelihood of CS using a free continuous confidence scale ranging from 0 to 100. The match rate of classification for ^18^F-FDG uptake patterns between cardiac and oncology NM physicians was evaluated by using the kappa coefficient.

### Model observer study

**Fig. 2:**
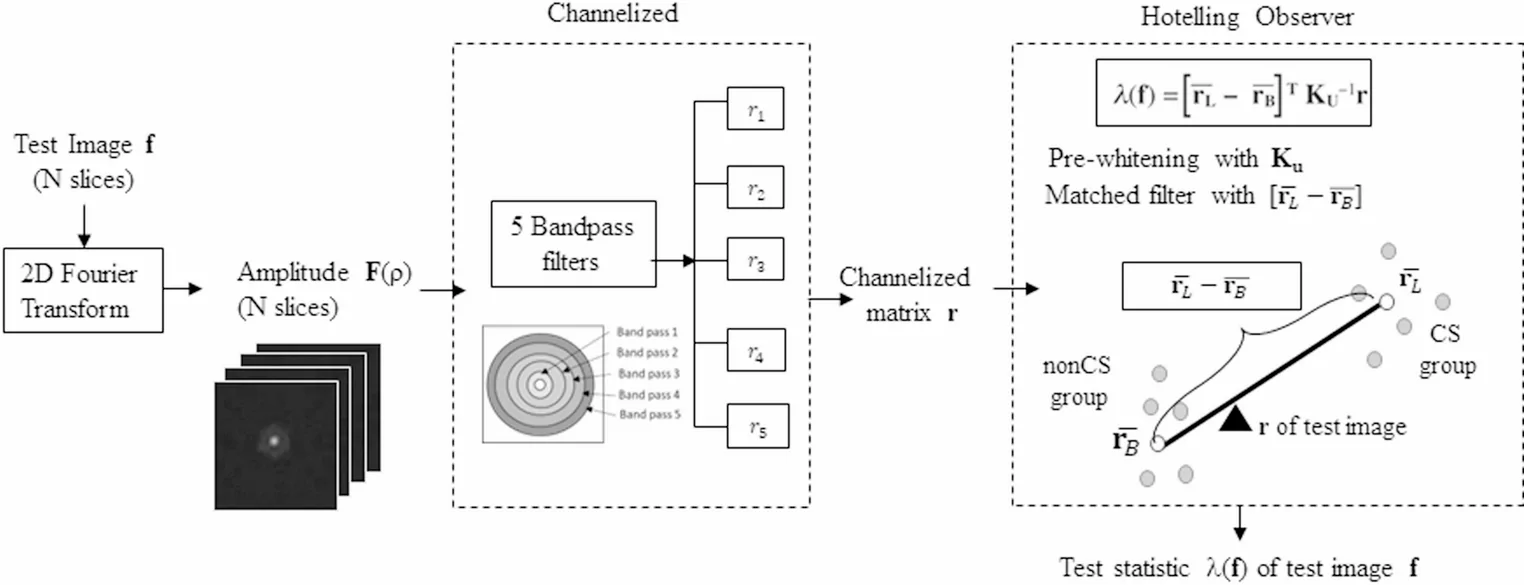
Workflow of CHO: After Fourier transforming test image **f** in each slice, channelized matrix **r** was generated by summing amplitude F over bandpass frequency and slices. Finally, test statistic λ(**f**) of test image **f** was calculated by operating channelized matrix **r** with pre-whitening and matched filter

To evaluate the objective discrimination ability of ^18^F-FDG uptake patterns in PET images, one of the model observers, CHO [25], was used. CHO attempts to use frequency features of image, where lower and higher frequency represents coarse and fine structures, respectively. As shown in Fig. 2, CHO accepts as an input an image *f* belonging to either a class of control images, f_B_, or a class of abnormal images (in this study, “abnormal” refers to CS), f_L_, and provides the test statistic λ, which indicates the statistical index that determines whether the input image f falls in one group or the other.

Initially, image f is converted by 2D Fourier transformation for each slice. In the frequency domain, bandpass filtering is applied for Fourier-transformed amplitude image F_n_(ρ) of slice *n* using radially symmetric channels u_c_(ρ) defined by the formula

1 $$\:{u}_{c}\left(\rho\:\right)=\left\{\begin{array}{c}1\:\lVert\rho\:\lVert\in\:\left[\left.{\rho\:}_{0}{2}^{c-1},{\rho\:}_{0}{2}^{c}\right)\right.\\\:0\:\:Otherwise\:\:\:\:\:\:\:\end{array}\right.\:\:\:\:$$

where for c ∈{1,2,3,4,5} and the frequency cut-off ρ_0_ was set to 0.03125 pixel^− 1^ in this study [26]. By this filtering process, the channel matrix r (= [r_1_,., r_c_]) is obtained as follows;

2 $$\:{r}_{c}\left(\rho\:\right)=\sum_{n=1}^{N}\frac{{\sum\:}_{\rho\:}{u}_{c}\left(\rho\:\right)\cdot \:{F}_{n}\left(\rho\:\right)}{{\sum\:}_{\rho\:}{u}_{c}\left(\rho\:\right)},$$

The test statisticλof a CHO for the task of detecting CS is as follows:

3 $$\:\lambda\:\left(f\right)={\left({\stackrel{-}{r}}_{L}-{\stackrel{-}{r}}_{B}\right)}^{T}{{K}_{U}}^{-1}\cdot\:r,$$

where r_L_ and r_B_ are the means of the two classes of channel matrices for the CS and control groups, respectively. The script “T” denotes a matrix transposition, and K_U_ is the c × c matrix averaged from the two-covariance matrices derived from channel matrices for the CS and control groups. For ROC analysis, test statistics λ for test data sets were normalized scale from 0 to 100 by using minimum and maximum λ values.

## Implementation of CHO

**Fig. 3:**
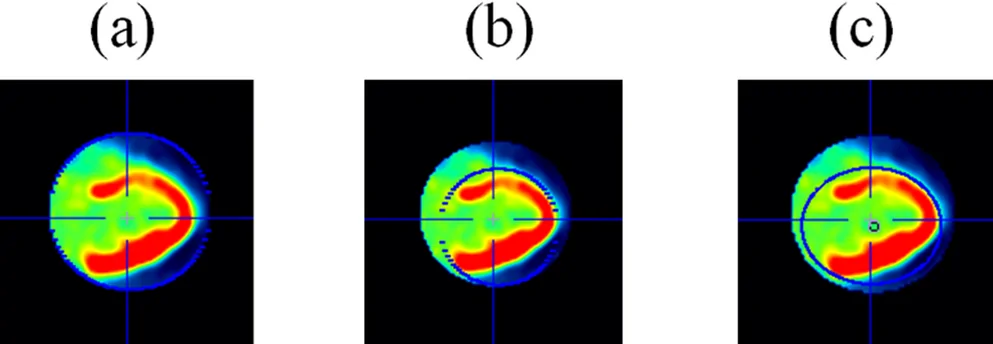
Three methods of VOI setting on the left ventricle for pixel-value normalization, (**a**) the sphere of 7 cm radius, (**b**) the sphere of 5.6 cm radius, and (**c**) the Elliptical Sphere surrounding the left ventricle myocardium

The volume ^18^F-FDG-PET images were resliced with short-, horizontal-, and vertical-axis, after determining the location of the left-ventricular apex and the angle of the axis. Spherical masking with a 7 cm radius (Fig. 3**(a)**) was used to eliminate outside regional uptakes such as the liver. For all subjects, a 2D Fourier transform was performed for the masked image in each slice, and then the Fourier-transformed image F(ρ) was summed over slices by Eq. (2).

All subjects were randomly classified into training and evaluation data sets. The training data set was 80 subjects (CS: 40, non-CS: 40) for deriving r_L_, r_B,_ and K_U_ in Eq. (3). Finally, test statistics of 143 subjects (CS: 66, non-CS: 77) were used to evaluate objective CS discrimination ability by CHO.

The effect of pixel values on CHO outcome was also investigated by normalizing from 0 to 100 based on SUV_max_ inside of manually placed 3 volume-of interest (7 cm, 5.6 cm radius, and the closest orbital elliptical sphere surrounding the left ventricle myocardium) (Fig. 3).

### Data analysis

Descriptive statistics were reported as mean ± standard deviation for parametric data and as counts for categorical data. The match rate of classified subject numbers between cardiac and oncology NM physicians were evaluated by the Kappa coefficient, where more than 0.8 as highly agreement, from 0.6 to 0.8 as quite agreement, from 0.4 to 0.6 as moderate agreement, and less than 0.4 as low agreement. The area under the curve (AUC), derived from receiver operating characteristic (ROC) analysis, was employed to assess the discriminatory capability of ^18^F-FDG uptake patterns in PET images for both human and CHO studies based on two groups (focal and focal-on-diffuse patterns as positive and diffuse and none as negative).

In the human interpretation study, a confidence scale ranging from 0 to 100 was determined by oncology NM physician for ROC analysis. In the CHO study, the test statistic λ in Eq. (3) was normalized to a scale ranging from 0 to 100 for ROC analysis.

The AUCs by human and CHO study were statistically compared by Pearson’s chi-square test. Welch’s t-test was employed to assess the means of parametric data, while Fisher’s exact test was utilized for the analysis of categorical data. A P value of less than 0.05 was considered statistically significant. All statistical analysis was performed using JMP pro (16.0.0, SAS Institute, Japan).

## Results

### Interpretation study by two physicians

Table 1 shows the characteristics of the CS and non-CS groups classified by cardiac NM physician. There were no significant differences in populations between the CS and non-CS groups.

Table 2 shows the independent classification of cardiac uptake in ^18^F-FDG-PET images by cardiac and oncology NM physicians without making any consensus. The match rate of classified subject numbers between cardiac and oncology NM physicians was moderate (Kappa coefficient resulted in 0.57), and then we defined cardiac NM physician as the reference of the classification in ROC analysis. The ratio of classified subject numbers between cardiac and oncology NM physicians were 23/31, 33/75, 50/68, and 46/49, in focal, focal on diffuse, diffuse, and none, respectively. In total 84 subjects were classified into different patterns, and the number of subjects that one physician classified as focal on diffuse but the other classified different patterns was about 50% of 84 subjects.

### ROC analysis with references to cardiac NM physician’s classification

**Fig. 4:**
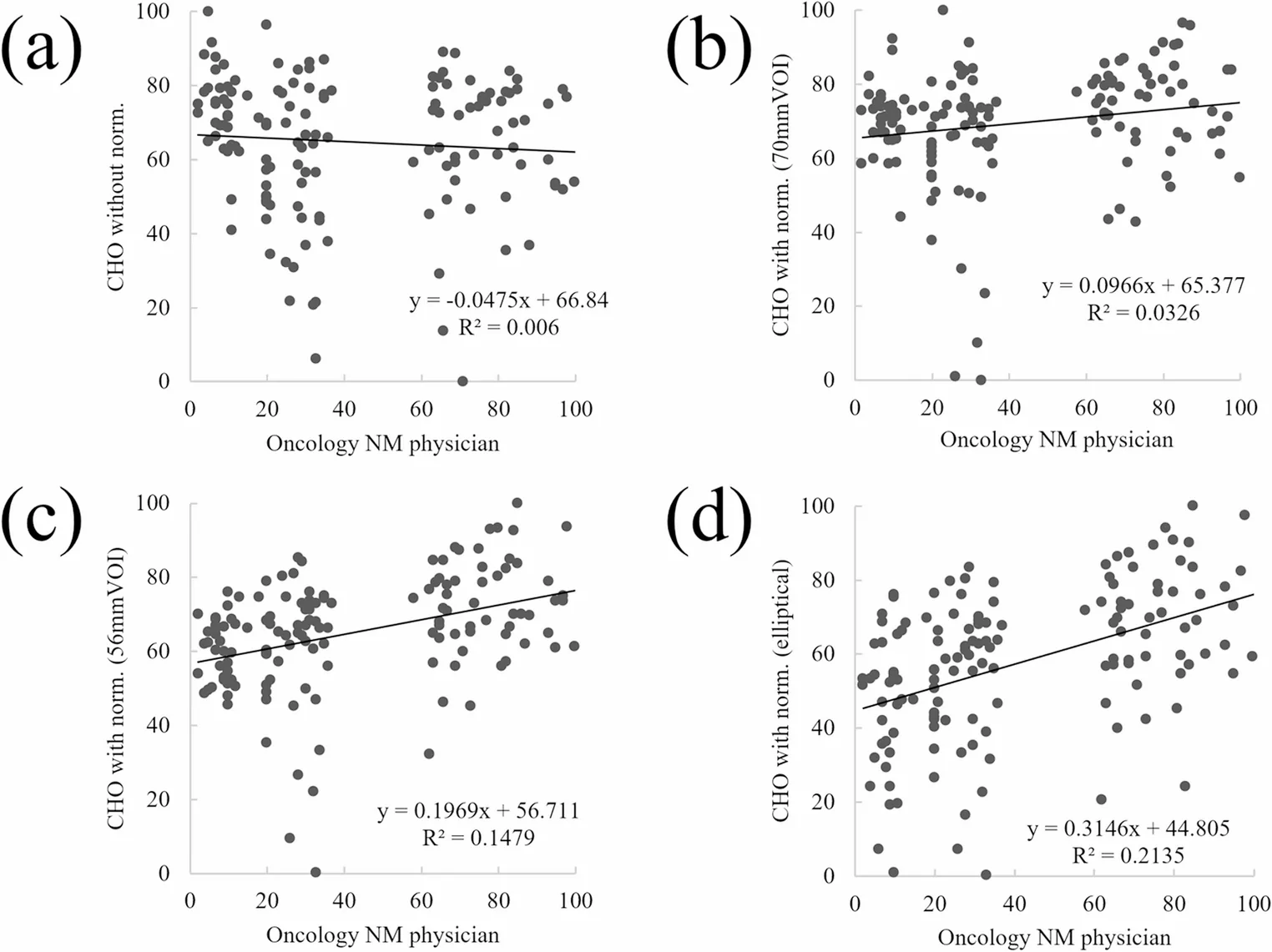
Fig. 4 Relationship of Rating scores by Oncology NM Physician and CHO with preprocessing, (**a**) 7 cm Spherical masking only, (**b**) Masking with normalization by 7 cm Spherical VOI, (**c**) Masking with normalization by 5.6 cm Spherical VOI and (**d**) Masking with normalization by Elliptical Sphere surrounding the left ventricle myocardium

**Fig. 5:**
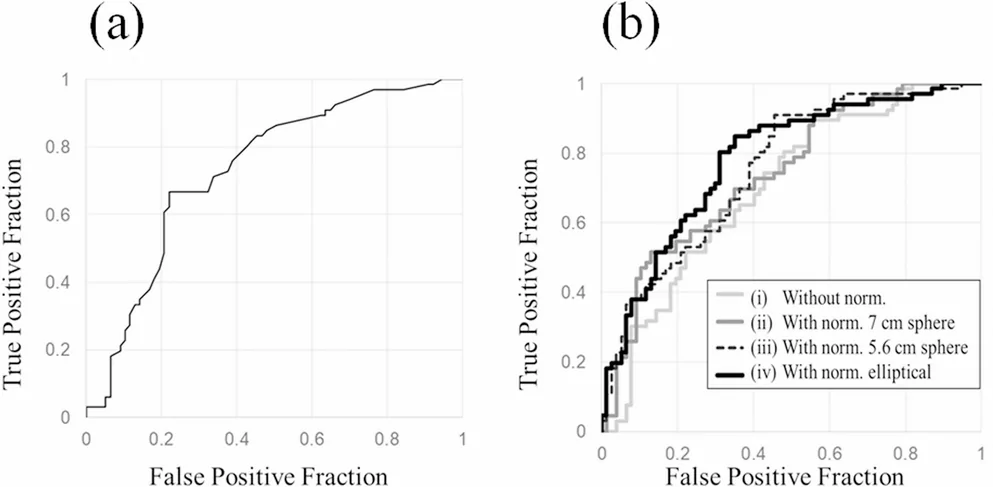
(**a**) ROC curve by oncology NM physician, (**b**) ROC curves by CHO with preprocessing, (i) 7 cm Spherical masking only, (ii) Masking with normalization by 7 cm Spherical VOI, (iii) Masking with normalization by 5.6 cm Spherical VOI and (iv) Masking with normalization by Elliptical Sphere surrounding the left ventricle myocardium

Figure 4 shows the relationship of rating scores by oncology NM Physician and CHO with 4 types of preprocessing. By introducing the normalization of pixel value and selecting the cardiac region, correlation coefficient of scores were improved. Figure 5; Table 3 show ROC curves and their AUC values by oncology NM physician and CHO with 4 preprocessing. The AUC values in CHO were improved from 0.70 to [0.74, 0.78] by the pixel-value normalization. The highest AUC of CHO was achieved with normalization of the elliptical sphere (0.78), and this was not a perfect match with cardiac NM physician (1.0), but consistent with the AUC of physician 2, 0.73. The reliability of CHO results was confirmed based on three-fold cross validation as shown in **Supplemental Table S1**.

## Discussion

Both visual and quantitative assessments have been performed to diagnose ^18^F-FDG-PET images of suspected CS patients. In this study, the CHO’s discrimination ability of healthy control and CS groups was compared to that of oncology NM physician. The consistency of CHO with cardiac expert NM physician was almost equivalent with the oncology NM physician. Our results suggested that the model observer, like CHO, has a potential alternative for assessing the discrimination ability of ^18^F-FDG uptake patterns in PET images by physicians in cardiac nuclear medicine.

### Interpretation study

The match rate of classification for ^18^F-FDG uptake patterns between cardiac and oncology NM physicians was moderate (kappa coefficient = 0.57), where this result was consistent with the previous study by Ohira et al., (kappa coefficient = 0.64) [8]. As shown in Table 2, there were trends of disagreement of classification for CS-patterns by cardiac and oncology NM physicians in the ventricular septum closer to the base of the heart and in the ventricular posterolateral wall between focal and focal on diffuse or focal on diffuse and diffuse. The site where disagreement of CS-pattern was the same as the site of a reported previous study [27]. Although cardiac and oncology NM physicians who participated in this study experiment have been involved in nuclear medicine for more than 20 years and both experienced nuclear medicine specialists, a high agreement rate of CS-pattern in PET images could not be obtained by visual assessment only [19]. These results suggest that the diagnosing of CS in PET images should be performed not only by visual assessment but also by quantitative assessment.

### Model observer study

It is known that CHO performance is influenced by acquisition protocol (orbit of SPECT cameras) and type of images (SPECT, 3D-SSP and Z-score) [20, 28]. Scan duration, reconstruction parameters, post-filtering, PSF-modeling, masking patterns and other factors are also well-known to influence image quality (diagnostic ability). The point of using CHO is the correlation of AUC with physicians, even though the image quality has been changed.

CHO requires an optimization scheme for every newly targeted disease or imaging modality. Visual classification of ^18^F-FDG-PET images between the CS and non-CS groups is a difficult task; therefore, pixel-value normalization, which may affect the difference in Fourier-transformed images F(ρ) between the CS and non-CS groups, was investigated. As shown in Figs. 3 and 5**(b)** and Table 3, the discrimination ability of CHO was dependent on the shape and size of the VOI for normalization. For “none” patterns of myocardial ^18^F-FDG-uptake, the location of SUV_max_ for normalization was expected to vary over the VOI. To distinguish between the CS and non-CS groups, selecting SUV_max_ values inside the cardiac region for all cases is important. In this study, the closest elliptical VOI was the best VOI for selecting the SUV_max_ values inside the cardiac region as shown in Fig. 4. Therefore, input PET images for CHO normalized by using the VOI surround shape of the myocardium showed the highest AUC values of 0.78.

In this study, discrimination ability by CHO was not significantly different from that of the human observer, as shown in Fig. 5. Thus, it was suggested that assessment for patterns using CHO would be useful as an alternative to pattern discrimination for PET images of CS patients by the cardiac NM physicians.

### Limitations of this study

This study has some limitations. In this study, the VOI setting of CHO for masking and normalization was performed by only one operator. Therefore, we could not evaluate inter-operator variability. Furthermore, because whole-body PET images were resampled using trilinear interpolation to the same voxel size of PET images in thoracic regions, their characteristic would not exactly match the original image characteristic. Sufficient reproducibility should be considered when performing preprocessing such as resampling and normalization in the future. Most severe limitations are that this study was based on the single reference by expert cardiac NM physician. This means ROC curves includes the bias caused from the diagnosis by a single physician and from selection of training data sets. Therefore, more patient data with positive cardiac biopsy as an absolute criterion for CS discrimination is required in the future.

Fasting condition of patients is also the critical limitation of this study. Our retrospective study only allowed ^18^F-FDG -PET data that patients fasted for at least 12 h. It is well-known that fasting time influences the uptake distribution in cardiac regions. Patients showed diffuse uptake and no patterns in this study may be influenced by the fasting time and effects on both pattern classification and interpretation study.

In this study, we have not made clinical diagnosis based on the JSSOG guideline [18] as the reference. This study focused on the diagnostic concordance in the cardiac ^18^F-FDG-PET uptake pattern of CHO and physician based on the reference of cardiac NM physician. For reference, the numbers of the patients diagnosed with CS based on JSSOG guideline are shown in **Supplemental Table S2.**

## Conclusion

The detection ability of CHO in CS pattern of myocardial ^18^F-FDG uptake was almost equivalent to that of the oncology NM physician when the diagnosis by the cardiac expert NM physician was defined as a reference standard. Even though the reference standard is not made by JCS guideline, the model observer has a potential alternative for assessing the discrimination ability of ^18^F-FDG uptake patterns of CS.

**Table 1:** Characteristics of CS group and non-CS group

	CS	non-CS	*p* value
	*n* = 106	*n* = 117	α = 0.05
Sex (male/female)	50/56	55/62	1.00
Age	58.9 ± 14.8	57.2 ± 13.9	0.38
BMI (kg/m2^)^	23.3 ± 4.3	23.3 ± 3.8	0.91
FBG [mg/dL]	99.5 ± 12.5	98.1 ± 4.3	0.43
Injection dose (MBq)	213.9 ± 4.6	216.3 ± 4.3	0.69

**Table 2:** Independent patters-classification of ^18^F-FDG PET images by two physicians (*n* = 223)

Cardiac NM physician	Oncology NM physician				total
	focal	focal on diffuse	diffuse	none	
focal	23	0	1	7	31
focal on diffuse	18	33	18	6	75
diffuse	1	16	50	1	68
none	1	1	1	46	49
total	43	50	70	60	223

**Table 3:** AUC values by Oncology NM physician and CHO

	AUC	[ 95% CI ]	*p* value
Oncology NM physician	0.73	0.64 - 0.81	-
CHO: (i)	0.70	0.61 - 0.78	0.61
CHO: (ii)	0.74	0.65 - 0.81	0.90
CHO: (iii)	0.75	0.67 - 0.82	0.66
CHO: (iv)	0.78	0.70 - 0.85	0.31

## Electronic Supplementary Material

Below is the link to the electronic supplementary material.

Supplementary material 1

Supplementary material 2
