# Supplementary material for: Detection ability of model observer for diagnosis of active cardiac sarcoidosis on 18F-FDG-PET
Source: Ann Nucl Med. 2026 Jun 15;40(11):1311–8. doi: 10.1007/s12149-026-02248-3 (PMC13683479; doi:10.1007/s12149-026-02248-3)
Supplement: Supplementary file 1 [file 12149_2026_2248_MOESM1_ESM.docx]

**Supplemental Table S1** Comparison of AUCs using CHO for VOI settings with an elliptical sphere surrounding the left ventricle myocardium

80 subjects (CS: 40, non-CS: 40) and 143 subjects (CS: 66, non-CS: 77) were used as the training and test datasets, respectively, in the original dataset split. In addition, two additional pairs of training and test datasets were constructed for 3-fold cross-validation. In additional dataset 1, the training dataset consisted of 71 subjects (CS: 30, non-CS: 41) and the test dataset consisted of 152 subjects (CS: 76, non-CS: 76). In additional dataset 2, the training dataset consisted of 72 subjects (CS: 36, non-CS: 36) and the test dataset consisted of 151 subjects (CS: 70, non-CS: 81). ~~Subjects were randomly assigned to each pattern.~~ Subjects with CS and non-CS were randomly assigned to the training and test datasets while maintaining the distribution of uptake patterns.
