# Supplementary material for: Detection ability of model observer for diagnosis of active cardiac sarcoidosis on 18F-FDG-PET
Source: Ann Nucl Med. 2026 Jun 15;40(11):1311–8. doi: 10.1007/s12149-026-02248-3 (PMC13683479; doi:10.1007/s12149-026-02248-3)
Supplement: Supplementary file 2 [file 12149_2026_2248_MOESM2_ESM.docx]

**Supplemental Table S2** Number of the patients diagnosed with Cardiac Sarcoidosis based on the Japanese Society of Sarcoidosis and Other Granulomatous Disorders (JSSOG) Criteria

Among the total 223 patients, 68 patients with CS pattern on FDG PET underwent a cardiac biopsy, of whom 9 patients were positive CS, and 58 patients with non-CS pattern on FDG PET underwent a biopsy, of whom 14 patients were positive. Based on the JSSOG criteria, 27 and 12 patients were clinically diagnosed with CS in the patients with CS and those with non-CS pattern on FDG PET, respectively.
